# Too packed to change: side-chain packing and site-specific substitution rates in protein evolution

**DOI:** 10.7717/peerj.911

**Published:** 2015-04-23

**Authors:** María Laura Marcos, Julian Echave

**Affiliations:** Escuela de Ciencia y Tecnología, Universidad Nacional de San Martín, San Martín, Buenos Aires, Argentina

**Keywords:** Protein evolution, Structural constraints, Packing, Contact density, Rate variation among sites, Side chain

## Abstract

In protein evolution, due to functional and biophysical constraints, the rates of amino acid substitution differ from site to site. Among the best predictors of site-specific rates are solvent accessibility and packing density. The packing density measure that best correlates with rates is the weighted contact number (WCN), the sum of inverse square distances between a site’s *C*_*α*_ and the *C*_*α*_ of the other sites. According to a mechanistic stress model proposed recently, rates are determined by packing because mutating packed sites stresses and destabilizes the protein’s active conformation. While WCN is a measure of *C*_*α*_ packing, mutations replace *side chains*. Here, we consider whether a site’s evolutionary divergence is constrained by main-chain packing or side-chain packing. To address this issue, we extended the stress theory to model side chains explicitly. The theory predicts that rates should depend solely on side-chain contact density. We tested this prediction on a data set of structurally and functionally diverse monomeric enzymes. We compared side-chain contact density with main-chain contact density measures and with relative solvent accessibility (RSA). We found that side-chain contact density is the best predictor of rate variation among sites (it explains 39.2% of the variation). Moreover, the independent contribution of main-chain contact density measures and RSA are negligible. Thus, as predicted by the stress theory, site-specific evolutionary rates are determined by side-chain packing.

## Introduction

Why do some protein sites evolve more slowly than others? Protein evolution is driven by random mutations and shaped by natural selection (Liberles et al., 2012; Sikosek & Chan, 2014). Mutations are selected depending on their impact on functional properties, such as the chemical nature of catalytic residues, active site conformation, and the protein’s ability to fold rapidly and stably. Since changes of these properties depend on the mutated site, amino acid substitution rates vary from site to site.

We can reformulate the question opening the previous paragraph: What *specific properties* account for site-dependent rates of evolution? The most studied predictors are structural site-specific properties (Franzosa & Xia, 2009). For years, the main structural predictor was believed to be *solvent accessibility*, as quantified by the Relative Solvent Accessibility (RSA) (Bustamante, Townsend & Hartl, 2000; Conant & Stadler, 2009; Franzosa & Xia, 2009; Ramsey et al., 2011; Shahmoradi et al., 2014). However, *local packing density*, quantified by the Weighted Contact Number (WCN), predicts evolutionary rates at least as well as RSA (Shih & Hwang, 2012; Yeh et al., 2014a; Yeh et al., 2014b).

The relationship between WCN and substitution rates can be understood in terms of a mechanistic stress model of protein evolution (Huang et al., 2014). Given an ancestral wild-type protein, the model assumes that its native conformation is the active conformation. Mutating a site perturbs (stresses) its interactions with other sites, destabilizing the active conformation. Such a destabilization determines the probability of the mutation being accepted or rejected, and therefore the rate of amino acid substitution. Using the energy function of the parameter-free Anisotropic Network Model (Yang, Song & Jernigan, 2009), the expected destabilization was found to be proportional to WCN, and site-specific substitution rates were predicted to decrease linearly with increasing WCN, in agreement with observations.

**Figure 1:**
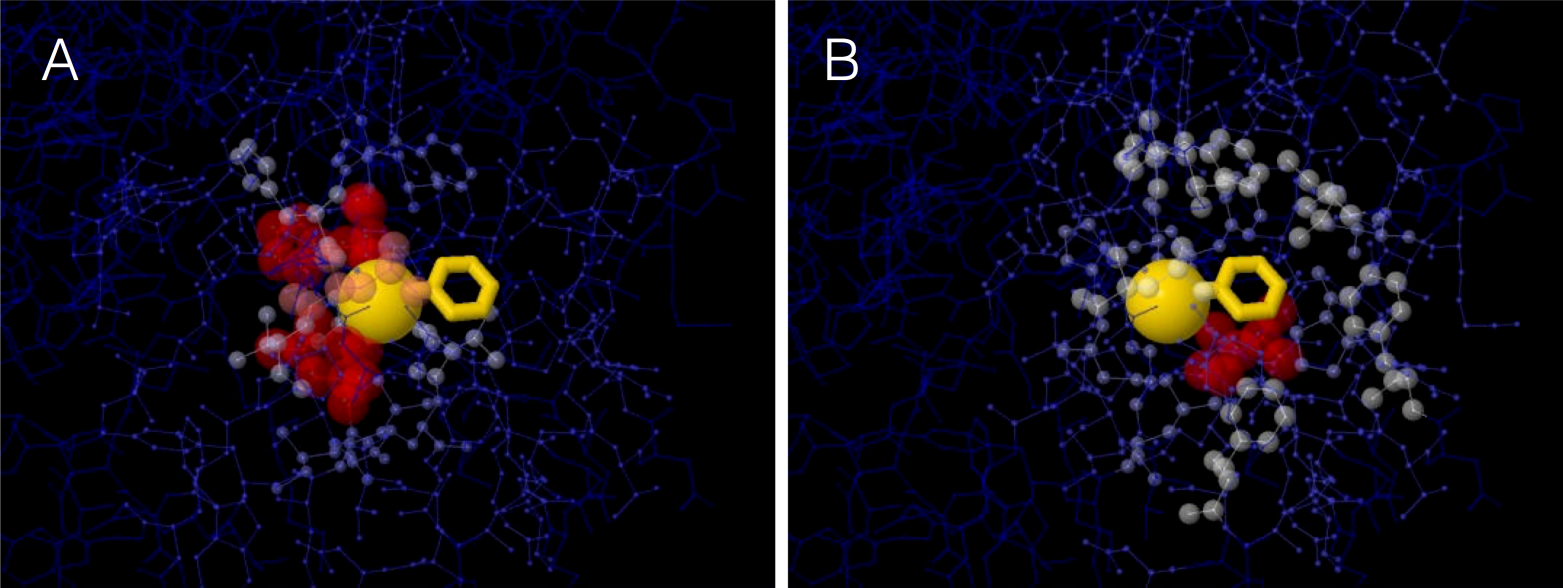
The two environments of a protein residue. Images of the environments of Thr93 of Human Carbonic Anhidrase II (pdb code 1CA2). (A) Environment of the main chain *C*_*α*_: the size and colors of protein atoms increase with the inverse square distance to Thr93 *C*_*α*_ (gold ball). (B) Environment of the side chain: size and colors of atoms increase with the inverse square distance to the geometric center of Thr93 side chain (gold wireframe).

A site’s WCN is the sum of inverse square distances from its *C*_*α*_ to the *C*_*α*_ of other sites: it is a measure of *C*_*α*_ packing density. Therefore, previous substitution rate vs. WCN studies were based on *main chain* (*C*_*α*_) packing (Shih & Hwang, 2012; Yeh et al., 2014a; Huang et al., 2014). However, mutations replace *side chains*. Consider a protein residue, e.g., Thr93 of Human Carbonic Anhidrase II (pdb code 1CA2) (Fig. 1). The environment of the main chain (Fig. 1A) differs from that of the side chain (Fig. 1B). When Thr93 is mutated, what environment would determine whether the mutation is accepted or rejected? More specifically: Do site-specific substitution rates depend on main-chain packing or on side-chain packing?

To address this issue, we extended the stress model to consider main and side chains explicitly and we theoretically derived that substitution rates depend only on side-chain of packing. We tested the theory on a data set of monomeric enzymes. In agreement with predictions, site-specific substitution rates correlate better with side-chain packing than with main-chain packing measures and RSA. Moroever, partialing out the effect of side-chain packing, the independent contributions of main-chain packing and RSA are negligible.

## Methods

### Theory

In this section, we show that the mechanistic stress model of protein evolution predicts that the substitution rate of a protein site is determined by the packing density of its side chain. This prediction and its empirical assessment are the point of this paper.

The stress model was proposed by Huang et al. (2014) to explain the observed correlation between site-specific substitution rates and packing density. The model is based on the idea that a mutant is viable to the extent that it spends time in the active conformation. In turn, this time will depend on mutational changes of the stability of the active conformation. The fixation probability of a mutant is modeled as

(1) \begin{eqnarray*} \displaystyle {p}_{\text{fix}}\propto \frac{{C}_{\text{mut}}^{F}{\rho }_{\text{mut}}({\mathbf{r}}_{\text{active}})}{{C}_{\text{wt}}^{F}{\rho }_{\text{wt}}({\mathbf{r}}_{\text{active}})}&&\displaystyle \end{eqnarray*}

where wt stands for wild-type, mut for mutant, *C^F^* is the concentration of folded protein and *ρ*(**r**_active_) its probability of adopting the active conformation. Assuming that *C*_mut_/*C*_wt_ is equal to the ratio of partition functions, from basic statistical physics it follows that:

(2) \begin{eqnarray*} \displaystyle {p}_{\text{fix}}\propto {e}^{-\beta \delta {V}^{\ast }},&&\displaystyle \end{eqnarray*}

where *β* represents the selection pressure and

(3) \begin{eqnarray*} \displaystyle \delta {V}^{\ast }={V}_{\text{mut}}({\mathbf{r}}_{\text{active}})-{V}_{\text{wt}}({\mathbf{r}}_{\text{active}})&&\displaystyle \end{eqnarray*}

is the energy difference between mutant and wild-type in the active conformation.

Assuming that *βδV*^∗^ ≪ 1 (weak selection), from (2) we find:

(4) \begin{eqnarray*} \displaystyle {K}^{i}\propto -\langle \delta {V{}^{\ast }\rangle }^{i},&&\displaystyle \end{eqnarray*}

i.e., the rate of substitution of site *i*, *K^i^*, is proportional to (minus) the change in stability of the active conformation averaged over mutations at *i*, 〈*δV*^∗^〉^*i*^. This is the basic equation of the stress theory.

In Huang et al. (2014), mutational stability changes were calculated using an elastic network model in which each residue is represented by a single node. Within such a one-node-per-residue representation, there is no differentiation between main chain and side chain. Therefore, we cannot *predict* whether evolutionary rates will be determined by main chain packing or side chain packing. To address this issue, here we represent each residue using *two nodes*: a main-chain node *α*, placed at the residue’s *C*_*α*_, and a side-chain node *ρ*, placed at the geometric center of the residue’s side chain (Gly’s are represented using only one node at *C*_*α*_). The energy function is:

(5) \begin{eqnarray*} \displaystyle V(\mathbf{r})&=&\displaystyle \frac{1}{2}\sum _{i}\sum _{j> i}{k}_{{\alpha }_{i}{\alpha }_{j}}({r}_{{\alpha }_{i}{\alpha }_{j}}-{d}_{{\alpha }_{i}{\alpha }_{j}})^{2}+\frac{1}{2}\sum _{i}\sum _{j> i}{k}_{{\alpha }_{i}{\rho }_{j}}({r}_{{\alpha }_{i}{\rho }_{j}}-{d}_{{\alpha }_{i}{\rho }_{j}})^{2}\nonumber\\ \displaystyle &&\displaystyle +\, \frac{1}{2}\sum _{i}\sum _{j> i}{k}_{{\rho }_{i}{\alpha }_{j}}({r}_{{\rho }_{i}{\alpha }_{j}}-{d}_{{\rho }_{i}{\alpha }_{j}})^{2}+\frac{1}{2}\sum _{i}\sum _{j> i}{k}_{{\rho }_{i}{\rho }_{j}}({r}_{{\rho }_{i}{\rho }_{j}}-{d}_{{\rho }_{i}{\rho }_{j}})^{2}, \end{eqnarray*}

where *r*_*n_i_n_j_*_ is the distance between nodes *n_i_* and *n_j_* (*n* is *α* or *ρ*), *k*_*n_i_n_j_*_ is the force constant of the spring connecting these nodes, and *d*_*n_i_n_j_*_ the equilibrium spring length.

A mutation at site *i* will replace *ρ_i_*, affecting only the parameters of the energy function related to this node. We emphasize: while the mutation may well induce global structural changes involving the backbone and other side chains, the only *parameters* that will change are those of the mutated side chain. Following Echave (2008) and Echave & Fernández (2010), we model a mutation at *i* by adding random perturbations to the lengths of the springs connected to *ρ_i_*: *d*_*ρ_i_ρ_j_*_ → *d*_*ρ_i_ρ_j_*_ + *δ*_*ρ_i_ρ_j_*_ and *d*_*ρ_i_α_j_*_ → *d*_*ρ_i_α_j_*_ + *δ*_*ρ_i_α_j_*_, to find, using (3) and (5):

(6) \begin{eqnarray*} \displaystyle \delta {V}^{\ast }=\frac{1}{2}\sum _{j\not = i}({k}_{{\rho }_{i}{\alpha }_{j}}{\delta }_{{\rho }_{i}{\alpha }_{j}}^{2}+{k}_{{\rho }_{i}{\rho }_{j}}{\delta }_{{\rho }_{i}{\rho }_{j}}^{2}).&&\displaystyle \end{eqnarray*}

Assuming that perturbations are drawn independently from the same distribution, averaging (6) over mutations at *i* we find:

(7) \begin{eqnarray*} \displaystyle \langle \delta {V{}^{\ast }\rangle }^{i}\propto \sum _{j\not = i}({k}_{{\rho }_{i}{\alpha }_{j}}+{k}_{{\rho }_{i}{\rho }_{j}}).&&\displaystyle \end{eqnarray*}

To finish, we assume, as in the parameter-free Anisotropic Network Model (pfANM) of Yang, Song & Jernigan (2009), that ${k}_{{n}_{i}{n}_{j}}=\frac{1}{{d}_{{n}_{i}{n}_{j}}^{2}}$. Then, from (4) and (7) we obtain:

(8) \begin{eqnarray*} \displaystyle {K}^{i}\propto -{\text{WCN}}_{\rho }^{\alpha \rho },&&\displaystyle \end{eqnarray*}

where

(9) \begin{eqnarray*} \displaystyle {\text{WCN}}_{\rho }^{\alpha \rho }=\sum _{j\not = i}\left(\frac{1}{{d}_{{\rho }_{i}{\alpha }_{j}}^{2}}+\frac{1}{{d}_{{\rho }_{i}{\rho }_{j}}^{2}}\right).&&\displaystyle \end{eqnarray*}

${\text{WCN}}_{\rho }^{\alpha \rho }$, derived here, is the side-chain weighted contact number. It depends on contacts between node *ρ* of the site considered (subscript) and nodes *α* and *ρ* of the other sites (superscript). Therefore, the stress model, combined with a two-nodes-per-site pfANM energy function, predicts that site-specific rates will depend on the contact density of the side chain ${\text{WCN}}_{\rho }^{\alpha \rho }$.

By analogy with (9) we can calculate the main-chain weighted contact number:

(10) \begin{eqnarray*} \displaystyle {\text{WCN}}_{\alpha }^{\alpha \rho }=\sum _{j\not = i}\left(\frac{1}{{d}_{{\alpha }_{i}{\alpha }_{j}}^{2}}+\frac{1}{{d}_{{\alpha }_{i}{\rho }_{j}}^{2}}\right).&&\displaystyle \end{eqnarray*}

We expect ${\text{WCN}}_{\alpha }^{\alpha \rho }$ to correlate with ${\text{WCN}}_{\rho }^{\alpha \rho }$, which may result in indirect correlations with substitution rates. However, if the stress model is correct, rates will be determined only by ${\text{WCN}}_{\rho }^{\alpha \rho }$ and there should not be any *independent* effect of ${\text{WCN}}_{\alpha }^{\alpha \rho }$.

### Other structural predictors

To assess the prediction of the previous section, we also consider the following structural properties. First, the Weighted Contact Number WCN, which was introduced by Lin et al. (2008) and found to be among the best structural predictors of site-dependent evolutionary rates (Yeh et al., 2014a; Yeh et al., 2014b). It is defined as:

(11) \begin{eqnarray*} \displaystyle \text{WCN}={\text{WCN}}_{\alpha }^{\alpha }=\sum _{j\not = i}\frac{1}{{d}_{{\alpha }_{i}{\alpha }_{j}}^{2}}&&\displaystyle \end{eqnarray*}

where *d*_*α_i_α_j_*_ is the distance between the the alpha carbons of sites *i* and *j*. For the sake of clarity, wherever it is convenient we will use the notation ${\text{WCN}}_{\alpha }^{\alpha }$ to make explicit that the distances between the *C*_*α*_ of a site (subscript) and the *C*_*α*_ of the other sites (superscript) are considered. Therefore, ${\text{WCN}}_{\alpha }^{\alpha }$ can be considered a measure of main-chain packing density (based only on *C*_*α*_–*C*_*α*_ interactions).

Second, by analogy with (11) we can use side-chain centers of mass *ρ* rather than *C*_*α*_ to define:

(12) \begin{eqnarray*} \displaystyle {\text{WCN}}_{\rho }^{\rho }=\sum _{j\not = i}\frac{1}{{d}_{{\rho }_{i}{\rho }_{j}}^{2}}&&\displaystyle \end{eqnarray*}

${\text{WCN}}_{\rho }^{\rho }$ quantifies the packing density of the side chain including only *ρ*–*ρ* interactions.

Finally, we also consider the Relative Solvent Accessibility, RSA, which is the most studied structural determinant of evolutionary rates. The RSA of a residue is obtained by dividing its area accessible to the solvent (SA) by the maximum SA for the given amino acid type (Tien et al., 2013).

### Dataset and empirical substitution rates

To test our theory, we used the data set of Echave, Jackson & Wilke (2015). The set consists of 209 monomeric enzymes of known structure covering diverse structural and functional classes. Each structure is accompanied by up to 300 homologous sequences.

We used the empirical site-specific rates of evolution of Echave, Jackson & Wilke (2015). They were calculated as follows. First, the homologous sequences for each structure were aligned using MAFFT (Multiple Alignment using Fast Fourier Transform) (Katoh et al., 2005; Katoh & Standley, 2013). Second, using the resulting alignments as input, Maximum Likelihood phylogenetic trees were inferred with RAxML (Randomized Axelerated Maximum Likelihood), using the LG substitution matrix (named after Le and Gascuel) and the CAT model of rate heterogeneity (Stamatakis, 2014). Third, the alignment and phylogenetic tree for each structure was used as input of Rate4Site to obtain the site-specific rates of substitution using the empirical Bayesian method and the amino-acid Jukes-Cantor mutational model (aaJC) (Mayrose et al., 2004). Finally, site-specific *relative* rates were obtained by dividing site-specific rates by their average over all sites of the protein. We denote the empirical rates by *K*_R4S_.

### Comparison of empirical rates with structural properties

For each protein of the dataset, we used the pdb structure to calculate the five site-dependent structural properties defined above: ${\text{WCN}}_{\rho }^{\alpha \rho }$, ${\text{WCN}}_{\alpha }^{\alpha \rho }$, ${\text{WCN}}_{\rho }^{\rho }$, ${\text{WCN}}_{\alpha }^{\alpha }$ (=WCN), and RSA. For a given predictor *x*, we quantified its predictive power using the squared Pearson correlation coefficient *R*^2^(*K*_R4S_, *x*). According to the theoretical predictions, ${\text{WCN}}_{\rho }^{\alpha \rho }$ should be the sole determinant of site-specific rates. We quantified the *independent* contribution of each of the other structural descriptors by partialing out the effect of ${\text{WCN}}_{\rho }^{\alpha \rho }$ using semipartial correlations. The squared semipartial correlation ${\rho }^{2}({K}_{\text{R4S}},x\vert {\text{WCN}}_{\rho }^{\alpha \rho })$ represents the *unique* contribution of predictor *x*. Also, it is the amount by which the explained variation of *K*_R4S_ (*R*^2^) would increase when going from the single-variable linear fit $K\sim {\text{WCN}}_{\rho }^{\alpha \rho }$ to the two-variable fit $K\sim {\text{WCN}}_{\rho }^{\alpha \rho }+x$. Expected values, standard deviations, and *p*-values were obtained by averaging protein correlations and semipartial correlations for 10,000 bootstrapped replicas of the dataset of 209 proteins.

For statistical analysis we used R (R Core Team, 2014). Correlation coefficients and their *p*-values were calculated using cor.test(). Semipartial correlation coefficients and *p*-values were calculated using spcor.test(). For bootstrapping with used boot() with default options.

## Results and Discussion

We theoretically derived a new measure of contact density, the side-chain weighted contact number ${\text{WCN}}_{\rho }^{\alpha \rho }$ which, according to the stress model, should be the sole structural determinant of site-specific evolutionary rates. We tested this prediction on a dataset of 209 functionally and structurally diverse monomeric enzymes. Empirical site-specific evolutionary rates *K*_R4S_ were obtained from multiple sequence alignments using Rate4Site. We compared *K*_R4S_ with ${\text{WCN}}_{\rho }^{\alpha \rho }$(side-chain weighted contact number), ${\text{WCN}}_{\alpha }^{\alpha \rho }$ (main-chain weighted contact number), ${\text{WCN}}_{\rho }^{\rho }$ (side-chain *ρ* − *ρ* weighted contact number), ${\text{WCN}}_{\alpha }^{\alpha }=\text{WCN}$ (main-chain *α* − *α* weighted contact number), and RSA (relative solvent accessibility). For each protein, we calculated correlation coefficients between *K*_R4S_ and each structural property and semipartial correlations to measure independent contributions. Protein-by-protein results (Table S1) were averaged over all proteins to obtain expected values, using bootstrapping to estimate standard deviations and p-values (see Methods).

### Side-chain contact density (${\text{WCN}}_{\rho }^{\alpha \rho }$) vs. main-chain contact density (${\text{WCN}}_{\alpha }^{\alpha \rho }$)

According to the stress model, site-specific substitution rates depend only on side-chain packing, so that main-chain packing should not be directly related to substitution rates. To test this prediction, we compared empirical substitution rates *K*_R4S_ with ${\text{WCN}}_{\rho }^{\alpha \rho }$ and ${\text{WCN}}_{\alpha }^{\alpha \rho }$.

**Figure 2:**
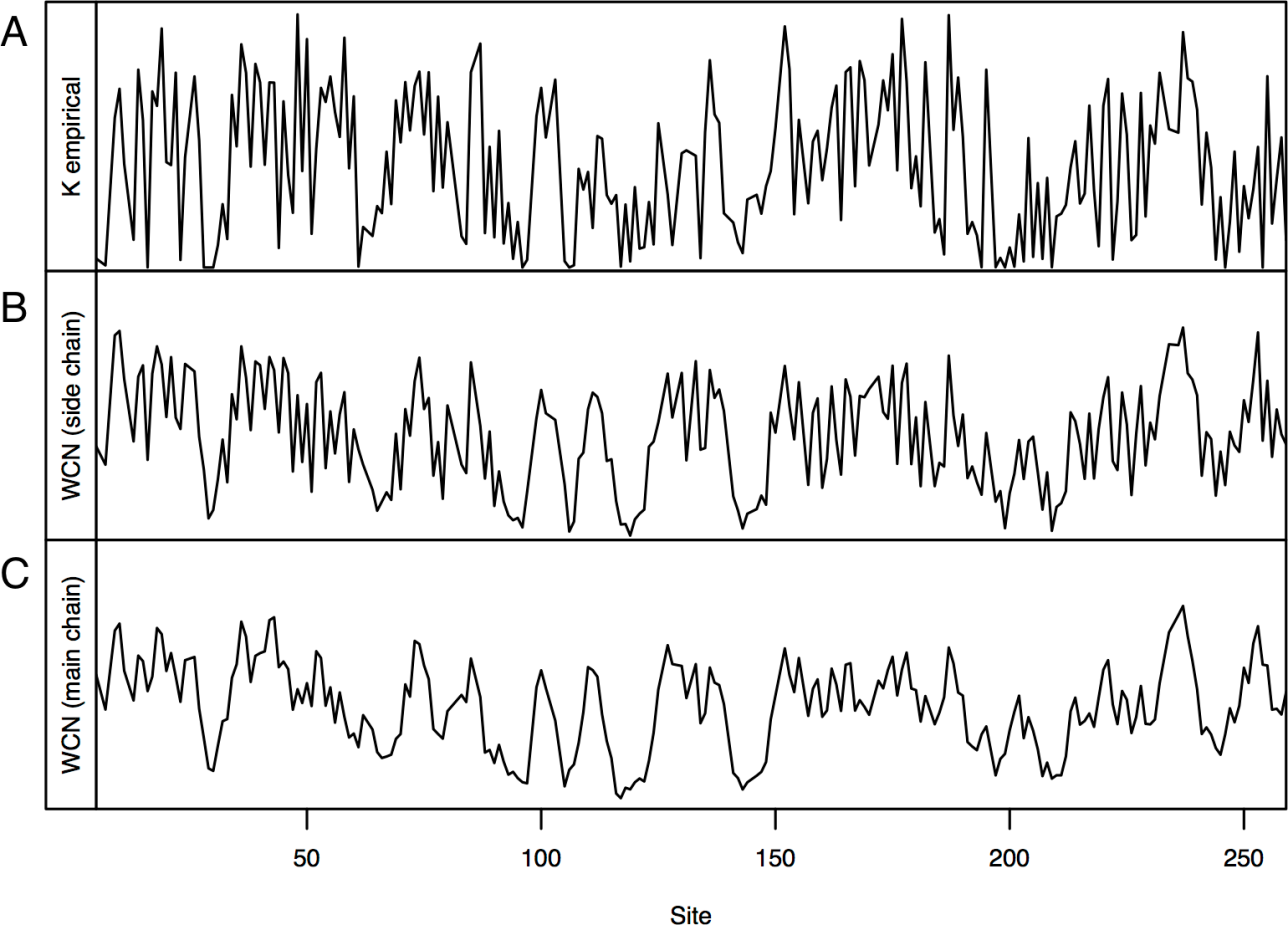
Profiles of site-specific evolutionary rates for 1CA2. (A) empirical rates *K*_*R*4*S*_ inferred by Rate4Site. (B) Rates predicted from the side-chain contact density $({\mathrm{WCN}}_{\rho }^{\alpha \rho })$. (C) Rates predicted from the main-chain contact density $({\mathrm{WCN}}_{\alpha }^{\alpha \rho })$. Both predicted profiles look similar to the *K*_R4S_ profile. However, the ${\mathrm{WCN}}_{\rho }^{\alpha \rho }$ profile is somewhat better (The ${\mathrm{WCN}}_{\alpha }^{\alpha \rho }$ profile is too smooth).

**Figure 3:**
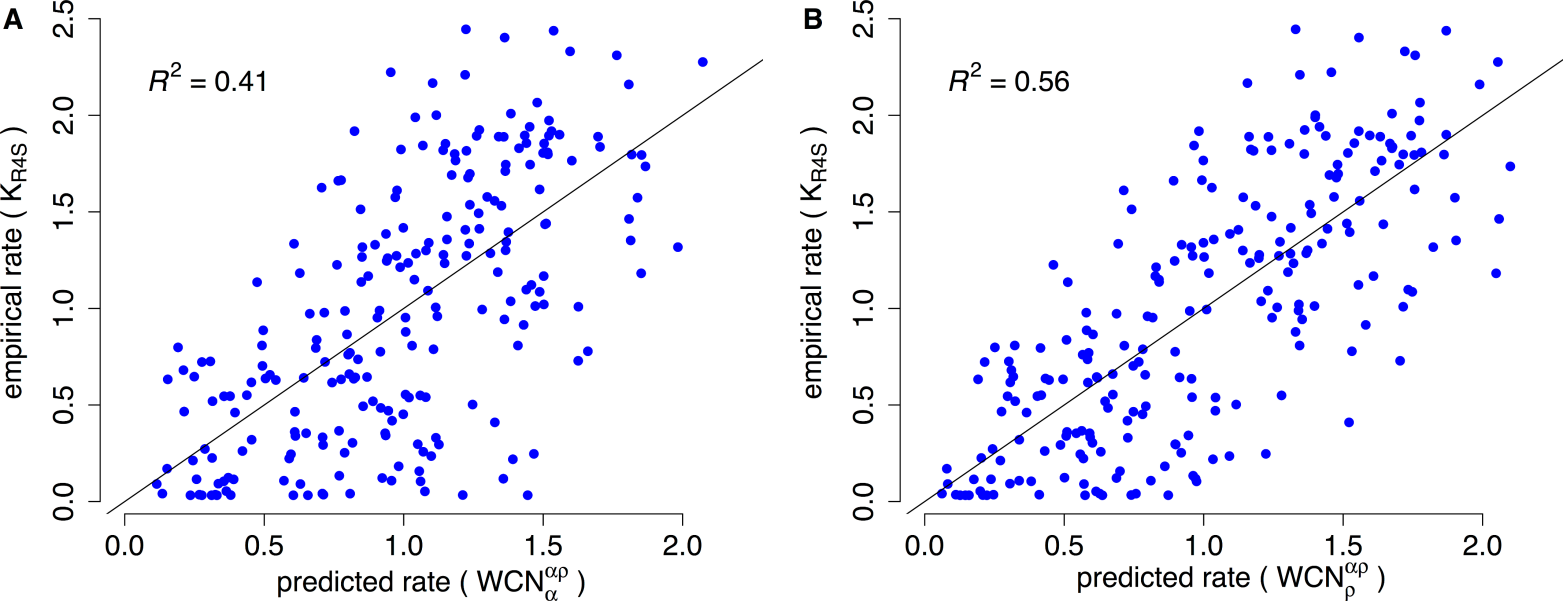
Empirical vs. predicted rates for 1CA2. (A) Empirical rates inferred using Rate4Site vs. rates predicted from the main-chain contact densities $({\mathrm{WCN}}_{\alpha }^{\alpha \rho })$ (B) Empirical rates vs. rates predicted from side-chain contact densities $({\mathrm{WCN}}_{\rho }^{\alpha \rho })$. The “*x* = *y*” line corresponding to a perfect fit is shown. ${\mathrm{WCN}}_{\alpha }^{\alpha \rho }$ explains *R*^2^ = 41% of the variation of site-specific empirical rates, ${\mathrm{WCN}}_{\rho }^{\alpha \rho }$ explains 56%.

Consider, for example, Human Carbonic Anhidrase II (pdb code 1CA2). As we mentioned in the Introduction, main chain environments and side-chain environments are different (Fig. 1). Accordingly, ${\text{WCN}}_{\rho }^{\alpha \rho }$ and ${\text{WCN}}_{\alpha }^{\alpha \rho }$ result in different predicted rates (Fig. 2). The two site-dependent profiles of predicted rates are similar to the empirical *K*_R4S_ profile. However, ${\text{WCN}}_{\rho }^{\alpha \rho }$-based predictions look somewhat better (Fig. 2) and are better (Fig. 3): the *R*^2^ values are 0.56 for ${\text{WCN}}_{\rho }^{\alpha \rho }$ and 0.41 for ${\text{WCN}}_{\alpha }^{\alpha \rho }$. Thus, for 1CA2 ${\text{WCN}}_{\rho }^{\alpha \rho }$ outperforms ${\text{WCN}}_{\alpha }^{\alpha \rho }$ as predictor of evolutionary rates.

**Figure 4:**
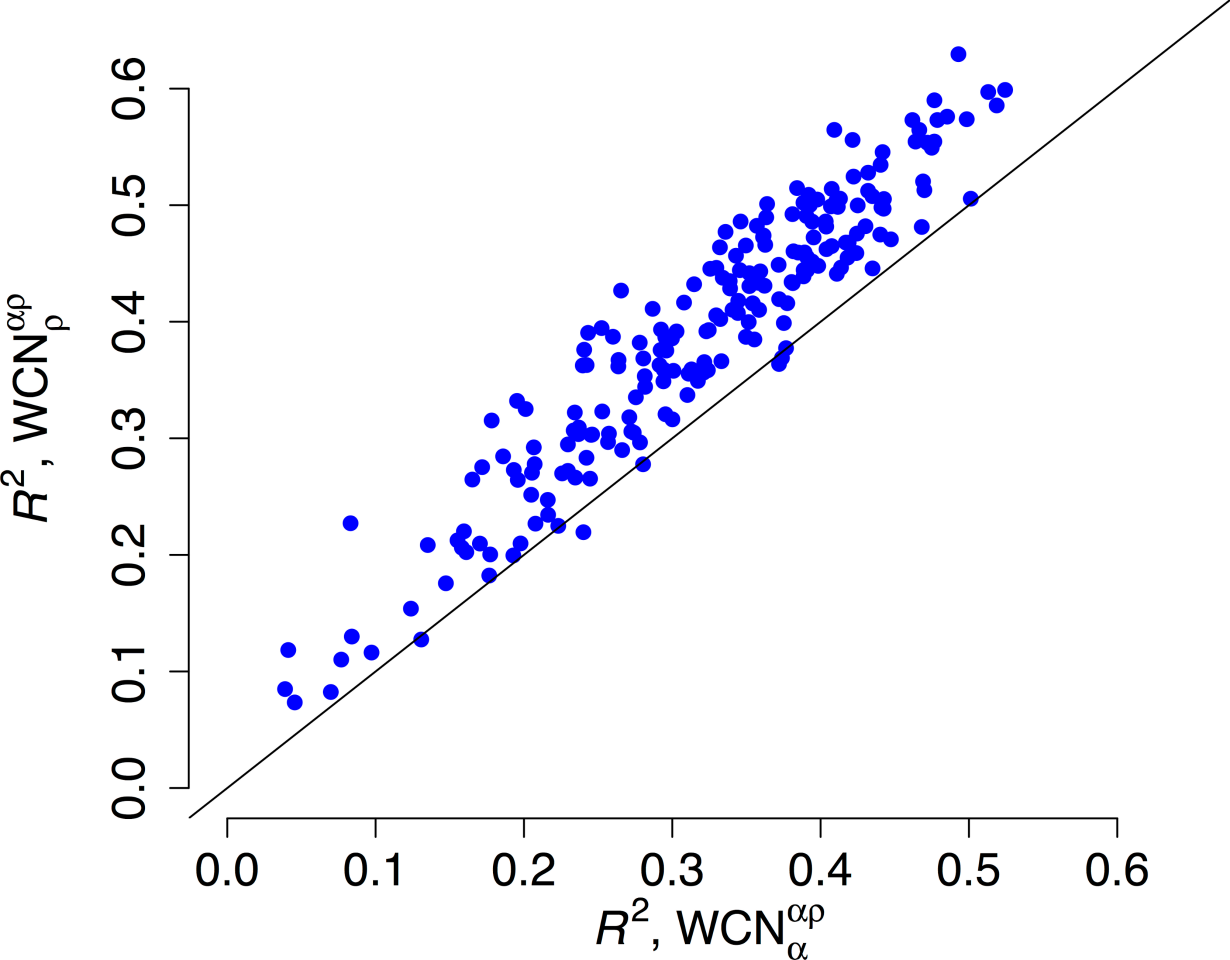
Side chain packing is the best predictor of substitution rates for most proteins. *R*^2^ is the square correlation between empirical rates (*K*_*R*4*S*_) and either side-chain contact density $({\mathrm{WCN}}_{\rho }^{\alpha \rho })$ (*y*-axis) or main-chain contact density $({\mathrm{WCN}}_{\alpha }^{\alpha \rho })$ (*x*-axis). Each point corresponds to one protein. Empirical rates correlate better with $({\mathrm{WCN}}_{\rho }^{\alpha \rho })$ for 204 out of 209 proteins.

We repeated the previous assessment for each of the 209 enzymes of the data set (Fig. 4). Empirical rates correlate with ${\text{WCN}}_{\rho }^{\alpha \rho }$ better than with ${\text{WCN}}_{\alpha }^{\alpha \rho }$ for 204 of the 209 proteins studied. Aggregating the data over all proteins, we obtained expected *R*^2^ values of 0.392 ± 0.008 and 0.319 ± 0.008 for ${\text{WCN}}_{\rho }^{\alpha \rho }$ and ${\text{WCN}}_{\alpha }^{\alpha \rho }$, respectively. The difference Δ*R*^2^ = 0.073 ± 0.003 is significantly positive (*p* < 10^−3^, bootstrapping). Therefore, ${\text{WCN}}_{\rho }^{\alpha \rho }$ outperforms ${\text{WCN}}_{\alpha }^{\alpha \rho }$ as predictor of site-specific substitution rates.

The stress model predicts ${\text{WCN}}_{\rho }^{\alpha \rho }$ to be the *sole* predictor of substitution rates. Any correlation between rates and ${\text{WCN}}_{\alpha }^{\alpha \rho }$ should be indirect. We measured the direct association between empirical rates and ${\text{WCN}}_{\alpha }^{\alpha \rho }$ using the squared semipartial correlation ${\rho }^{2}({K}_{\text{R4S}},{\text{WCN}}_{\alpha }^{\alpha \rho }\vert {\text{WCN}}_{\rho }^{\alpha \rho })$, where the variation of rates due to ${\text{WCN}}_{\rho }^{\alpha \rho }$ is partialed out. This measure is the *unique* contribution of ${\text{WCN}}_{\alpha }^{\alpha \rho }$ and it represents how much *R*^2^ would increase when going from the one variable model $K\sim {\text{WCN}}_{\rho }^{\alpha \rho }$ to the two-variable model $K\sim {\text{WCN}}_{\rho }^{\alpha \rho }+{\text{WCN}}_{\alpha }^{\alpha \rho }$. Averaging over the 209 proteins studied, we found ${\rho }^{2}({K}_{\text{R4S}},{\text{WCN}}_{\alpha }^{\alpha \rho }\vert {\text{WCN}}_{\rho }^{\alpha \rho })=0.0024\pm 0.0005$. This value is statistically significant (*p* < 10^−3^, bootstrapping), but *very small*: ${\text{WCN}}_{\alpha }^{\alpha \rho }$’s unique contribution to rate variation among sites is just 0.2%. As predicted by the stress model, the independent contribution of ${\text{WCN}}_{\alpha }^{\alpha \rho }$ is negligible.

### ${\text{WCN}}_{\rho }^{\alpha \rho }$ vs. ${\text{WCN}}_{\rho }^{\rho }$

${\text{WCN}}_{\rho }^{\alpha \rho }$, Eq. (9), is based on a two-nodes-per-site network representation of the protein. It considers the contacts between the node *ρ* that represents the side chain of a site with all other nodes, *ρ* and *α* of the network. ${\text{WCN}}_{\rho }^{\rho }$, Eq. (12), is an alternative alternative measure of side-chain packing based only on *ρ* − *ρ* contacts. ${\text{WCN}}_{\rho }^{\alpha \rho }$ is a better rate predictor than ${\text{WCN}}_{\rho }^{\rho }$ for 122 of the 209 proteins. The expected correlations are ${R}^{2}({K}_{\text{R4S}},{\text{WCN}}_{\rho }^{\alpha \rho })=0.392\pm 0.008$ and ${R}^{2}({K}_{\text{R4S}},{\text{WCN}}_{\rho }^{\rho })=0.389\pm 0.008$. The average difference Δ*R*^2^ = 0.0024 ± 0.007 is significant (*p* < 10^−3^, bootstrapping), but very small (just 0.24% of explained variation). Thus, ${\text{WCN}}_{\rho }^{\rho }$-based predictions are (almost) as good as ${\text{WCN}}_{\rho }^{\alpha \rho }$ predictions. However, while ${\text{WCN}}_{\rho }^{\rho }$ was posed *ad hoc*, ${\text{WCN}}_{\rho }^{\alpha \rho }$ was *theoretically derived*.

### ${\text{WCN}}_{\rho }^{\alpha \rho }$ vs. WCN

Currently, WCN ($={\text{WCN}}_{\alpha }^{\alpha }$), the original weighted contact number (Lin et al., 2008), is one of the two main structural predictors of site-dependent evolutionary rates (Yeh et al., 2014a; Yeh et al., 2014b). It is worthwhile to consider whether the new measure presented here, ${\text{WCN}}_{\rho }^{\alpha \rho }$ provides an improvement over WCN.

We found that ${\text{WCN}}_{\rho }^{\alpha \rho }$ outperforms WCN for 206 out of the 209 proteins studied. The expected correlations are ${R}^{2}({K}_{\text{R4S}},{\text{WCN}}_{\rho }^{\alpha \rho })=0.392\pm 0.008$ and *R*^2^(*K*_R4S_, WCN) = 0.314 ± 0.007. The difference is Δ*R*^2^ = 0.078 ± 0.003, which is statistically significant (p ≪ 10^−3^, bootstrapping). Thus, not only does ${\text{WCN}}_{\rho }^{\alpha \rho }$ outperform WCN for almost all proteins, but by a rather large amount: while WCN explains 31.4 % of the variation of evolutionary rates, ${\text{WCN}}_{\rho }^{\alpha \rho }$ explains 39.2%, an increase by a factor of 1.25. Moreover, ${\rho }^{2}({K}_{\text{R4S}},\text{WCN}\vert {\text{WCN}}_{\rho }^{\alpha \rho })=0.0024\pm 0.005$ (p < 10^−3^, bootstrapping). Despite statistical significance, the unique contribution of WCN is just 0.2%, which is negligible. Thus, ${\text{WCN}}_{\rho }^{\alpha \rho }$ is a better predictor and the independent contribution of WCN is negligible.

### ${\text{WCN}}_{\rho }^{\alpha \rho }$ vs. RSA

The most studied structural predictor of site-dependent evolutionary rates is the relative solvent accessibility RSA (Bustamante, Townsend & Hartl, 2000; Conant & Stadler, 2009; Franzosa & Xia, 2009; Ramsey et al., 2011; Shahmoradi et al., 2014). Therefore, we compare the new measure ${\text{WCN}}_{\rho }^{\alpha \rho }$ with RSA.

According to protein-by-protein results, ${R}^{2}({K}_{\text{R4S}},{\text{WCN}}_{\rho }^{\alpha \rho })> {R}^{2}({K}_{\text{R4S}},\text{RSA})$ for 175 of the 209 proteins. The expected square correlations are ${R}^{2}({K}_{\text{R4S}},{\text{WCN}}_{\rho }^{\alpha \rho })=0.392\pm 0.008$ and *R*^2^(*K*_R4S_, RSA) = 0.327 ± 0.007. The difference is Δ*R*^2^ = 0.065 ± 0.004, which is statistically significant (*p* < 10^−3^, bootstrapping). Thus, ${\text{WCN}}_{\rho }^{\alpha \rho }$ outperforms RSA as rate predictor for 84% of the proteins and ${\text{WCN}}_{\alpha }^{\alpha }$ explains 6.5% more of the rate variation among sites, an improvement by a factor of 1.2 over the explaining power of RSA. Moreover, the expected value of the independent contribution of RSA is $\rho ({K}_{\text{R4S}},\text{RSA}\vert {\text{WCN}}_{\rho }^{\alpha \rho })=0.005\pm 0.001$). This is statistically significant (*p* < 10^−3^, bootstrapping), but very small. Therefore, ${\text{WCN}}_{\rho }^{\alpha \rho }$ is a better predictor and the independent contribution of RSA is minor.

## Conclusion

We used the the mechanistic stress model to predict theoretically that site-specific rates of evolution depend solely on the side-chain contact density ${\text{WCN}}_{\rho }^{\alpha \rho }$. According to the stress theory, ${\text{WCN}}_{\rho }^{\alpha \rho }$ is proportional to the mutational destabilization of the protein’s active conformation, which is why it correlates with rates: mutations are accepted or rejected according to the degree of destabilization. We tested this prediction on a large dataset of monomeric enzymes. We found that ${\text{WCN}}_{\rho }^{\alpha \rho }$ outperforms ${\text{WCN}}_{\rho }^{\rho }$, WCN and RSA and that the independent contributions of the latter are negligible, which supports the theoretical prediction.

To finish, we note that the structural properties studied do not explain all of the variation of substitution rates among sites. The best predictor, ${\text{WCN}}_{\rho }^{\alpha \rho }$ explains on average ∼39% of the variation, leaving 61% unexplained. Further research is needed to gain a full understanding of the variation of substitution rates among protein sites.

## Supplemental Information

10.7717/peerj.911/supp-1 Table S1 Correlation coefficients for all proteins.
